# Enduring rules of care within pairs - how blue tit parents resume provisioning behaviour after experimental disturbance

**DOI:** 10.1038/s41598-019-39139-9

**Published:** 2019-02-26

**Authors:** Arne Iserbyt, Maaike Griffioen, Marcel Eens, Wendt Müller

**Affiliations:** Behavioural Ecology and Ecophysiology Group, University of Antwerp, Universiteitsplein 1, B-2610 Wilrijk, Belgium

## Abstract

Sexual conflict over parental investment can result in suboptimal reproductive output. A recent hypothesis suggests that equality in investment, and hence conflict resolution, may be reached via coordination of parental activities like alternating nest visits. However, how robust patterns of care within couples are against temporal disturbances that create asymmetries in parental investment remains as yet to be shown. We here experimentally created such a social disturbance in a wild population of biparental blue tits (*Cyanistes caeruleus*) when provisioning their nestlings. We randomly caught and subsequently released one of the parents when nestlings were 6 and 12 days old respectively. On average, the parent that was caught did not resume care for nearly two hours. We then compared the levels of individual investment and within-pair coordination before, during and after the absence of the disturbed parent. We show that the remaining parent partially compensated by increasing its provisioning rate, but this compensatory response was strongest in females when nestlings were 6 days old. Once the caught parent returned to feed its nestlings, both parents resumed provisioning at a similar rate as before the disturbance. Likewise, the within-pair alternation level quickly resembled the pre-manipulated level, independent of nestling age or which sex was caught. Thus our experiment highlights the resilience of parental behaviour against temporal disturbances of individual parents.

## Introduction

Cooperative behaviour, i.e. the simultaneous or consecutive acting together of two or more individuals^1^, often yields mutual benefits. It is taxonomically widespread and persisted over long periods of evolutionary time^2^. Insect eusociality^3^, cooperative breeding in birds^4^ and coalition formation in mammals^5^ are just a few examples demonstrating the multiplicity of cooperative behaviour that can be observed. These examples usually involve interactions among relatives, supporting the general belief that cooperation has evolved and is maintained through kin selection. However, this interpretation has been challenged by the observation that cooperative interactions among non-relatives frequently occur in a variety of contexts^6–8^. A comprehensive understanding of the evolutionary mechanisms promoting cooperative behaviour among kin and non-kin is thus still open for discussion^1^.

One of the most common forms of cooperation among non-relatives is biparental care, in which two unrelated individuals join, at least temporarily, to care for their offspring^9^. Meanwhile, however, family life forms a battleground, rife with individual strategies and conflicts among all family members^10,11^. Fundamental to the conflict between parents are the costs that arise from the time, energy and resources needed to invest into their current offspring, at the expense of investment in future offspring. Each parent profits from any investment into parental care, while parents only pay the costs of their own contribution^12^. Despite these diverging interests, fitness benefits of collaboration often outweigh the costs that would be associated with one parent abandoning the offspring^13^. Thus, given the benefits of biparental care, reproductive strategies have evolved to transcend sexual conflict and to facilitate parental cooperation. Recent evidence suggests that not only the total amount of each parents’ investment matters^14^, but that also the timing of distinct bouts of investment could be crucial for parents to maximize reproductive output (e.g. via nest visit alternation^15^ or nest visit synchronization^16^). Cooperation thus potentially involves the coordination of activities between pair members, which inherently implies a constant monitoring and corresponding behavioural adjustments towards each other’s activities (i.e. ‘negotiation’)^17–19^.

Several empirical studies have found that individuals match their partners’ effort^20–22^, which inspired the development of a theoretical model for conflict resolution, based on a simple rule of direct reciprocity^15^. That is, each parent should speed up its feeding rate after a recent visit of the partner, but wait for the partner to feed if the individual itself was last to visit the nest. Such flexibility in the rate and coordination of nest visits may translate into alternated nest visits in which parents systematically take turns when feeding their offpring^15,23–25^. Such a pattern of alternation may prevent substantial free-riding on the effort of the partner, because low investment is punished through the matched response of the partner. Even though it can be assumed that alternation is not free of faulty decisions, imperfect alternation may still ease the sexual conflict over parental care. However, this hypothesis has also been subject to criticism. Alternated bouts of investment require that both parents share a task like incubation or offspring provisioning, which is not generally true across-^26^ and even within species^27^. It has also been argued that parental turn-taking does not reflect an active behavioural strategy but that it arises from spatio-temporal correlations of foraging under common conditions, i.e. when the time between visits is similar for both parents and correlated through time^28^. Thus, whether alternation reflects either active co-adjusted investment between pair members, or rather an analogue for individual feeding strategies is still open for debate. These possibilities may be difficult to disentangle, but a fruitful step forward may require detailed behavioural studies or experimental approaches focusing on the consistency of feeding behaviour. However, experiments that aim to investigate the level of parental care on both the individual and the pair level under experimentally manipulated conditions remain very rare^18,29^.

The aim of this study was therefore to experimentally test the resilience of both absolute and coordinated levels of parental investment, by temporarily disturbing within-pair coordination of parental activities. To this end, we caught one of both blue tit (*Cyanistes caeruleus*) parents and released it back into its own territory soon thereafter. A recent study showed that once caught, blue tits stay within the proximity of their nest, but on average do not participate in nestling feeding for almost two hours^30^. Most importantly, this procedure differs from experimental handicapping or widowing^14^, as the condition of the manipulated bird remains unaltered. It resembles a natural situation in which one of the parents is challenged by, for example, a predator or an intruder. Comparing partners in their absolute levels of investment (e.g. feeding rates), as well as their coordinated levels of investment (e.g. alternation) before, during and after the disturbance, enabled us to determine to which extent these behavioural estimates are flexible and whether they potentially became re-established over time. Our disturbance was performed at two different stages of offspring need, i.e. with six days old poikilothermic and twelve days old endothermic nestlings^31^, to investigate the consequences of dynamic asymmetries in parental tasks for cooperation^23^. Brooding is a distinct female task which makes them well informed and potentially more responsive to environmental changes^32^. We are particularly interested in the moment that the disturbed individual resumed parental care, how the lacking contribution to care of the formerly absent parent impinges on its own and its partner’s feeding behaviour. We expect that pair members monitor each other’s activities and speed up or slow down nest visit rate in direct response of the partner’s behaviour, as would be predicted by the alternation hypothesis^15^. We expect that this matching response is an efficient way to achieve equality in investment, which would prevent any further exploitation by the formerly absent parent.

## Methods

### Study system

Fieldwork was performed in two consecutive years (2016 and 2017) between March and May in a nest box population of blue tits breeding in Peerdsbos, a mature oak-beech forest near Antwerp (N51° 16′, E4° 28′, Belgium)^33^. Nest boxes (N = 131) were checked twice per week for nest building, egg laying and incubation. From the expected hatch date nests were checked daily until hatching, here defined as day 0.

### Behavioural activity

To quantify individual behavioural activity, we made use of 2.6 mm plastic leg bands with passive integrated transponder (PIT) tags (EM4102, 125 KHz, Eccel Technology Ltd, Aylesbury, U.K). These tags were fitted around the legs while blue tits were caught in the nest box either when roosting in winter, during incubation (females) or during preceding breeding seasons, but not during the nestling phase of this study. This was done because banding itself significantly increases nest return time^30^, which potentially interferes with our behavioural observations (see further). Circular radio-frequency-identification (RFID) antennas connected to a logger were installed around the front of the nest box opening and continuously registered parental nest visits until fledging. Installing the antennas was done in less than two minutes, which ensured minimal human disturbance. The equipment was installed when nestlings were 1–5 days old, enabling at least one day of habituation prior to the experiments. All birds accepted this modification at their nest box and resumed provisioning behaviour after 17.9 ± 2.8 min (range: 2.2–98.9 min). A full description of the RFID hardware, analytical procedure and data validation is detailed in Iserbyt *et al*.^34^. In short, exact arrival times were isolated from successive redundant RFID registrations, by sorting all registrations per ID and erasing each registration within 23 seconds after the preceding registration^34^. Based on these trimmed readings we calculated individual behavioural activity as the number of nest arrivals for each hour (i.e. nest visit rate). Nest visit rate is frequently interpreted as an estimate for individual investment in offspring provisioning^9^, which has been confirmed for our study species^34^. We then sorted the trimmed readings chronologically to calculate the proportion of alternated nest visits. That is when a male visit follows a female visit and vice versa, and is interpreted as an estimate for the level of coordinated provisioning within a pair^15^. The level of alternation was calculated as the number of alternated male and female visits, relative to the total number of nest visits, minus one^24^. Both parameters, individual nest visit rate and pair alternation scores, have previously been validated thoroughly by calibrating RFID data against video recordings (range correlation coefficients, r = 0.88–0.90)^34^. More detailed, every single food delivery in an analyzed video fragment was aligned with the processed RFID registrations, which revealed that 6.8% of the ‘true’ visits were overrated (touching but not passing through the antennas or spending >23 s in the nest box) and 15.7% were not registered (very fast passage or nest return within 23 s).

### Experimental protocol

To temporarily disturb parental feeding patterns, we randomly caught either the male or the female. This was done around noon (mean ± SD: 12 h 42 ± 1 h 05) on two specific days, i.e. day 6 and day 12 of the nestling period. Birds were caught by rapidly (<10 sec) blocking the nest box entrance with a metal plate mounted on a wooden stick after the bird had entered the nest box. When captured, a series of standard measurements were taken, at least 15 meters away from the nest box. These measurements included blood (~50 μl) and feather (2^nd^ outer tail) sampling, measuring body mass and tarsus length. None of the birds included in this study were banded for the first time, as this has been shown to significantly increase the nest return time^30^. Birds were put in a cloth bag until a total of 15 minutes had passed from capture and subsequently released in their territory. This protocol enabled us to divide an experimental day in three distinct parts (time windows). Part A is referred to as ‘Control’ throughout the manuscript and corresponds with the period between 8 am and the time of capture (mean ± SD time period: 4 h 42 ± 1 h 05). Part B is called ‘Alone’ and refers to the period between the nest return of the remaining parent and the nest return of the caught parent (1 h 26 ± 1 h 10). Part C is called ‘Reunion’, which refers to the period between the return of the caught parent and 6 pm (3 h 56 ± 1 h 28). On day 6 (D6) and day 12 (D12), we caught respectively 18 and 17 males, and 14 and 20 females. Both partners were caught in 15 nests, though on different experimental days. In total, we quantified behavioural activity from 54 unique nests in which both parents were tagged throughout an entire experimental day (i.e. part A–C). In addition, we quantified any potential diurnal variation in visit rates and alternation scores on two days without any human disturbance (i.e. day 7 and day 13). Due to the limited number of available RFID systems, among other logistical constrains, our sample size on day 5 and day 11 was too low to use as reference days. The behavioural parameters on both reference days were calculated between 8–12 am (‘Control’), 12 am–2 pm (‘Alone’) and 2–6 pm (‘Reunion’), i.e. the three time windows that correspond with those on the experimental days.

### Compliance with ethical standards and data availability

Animal use and experimental design were approved by the Ethical Committee of the University of Antwerp, Belgium (ID: 2012–51 and 2015–64). All experiments were carried out in accordance with the FELASA (Federation of European Laboratory Animal Science Associations) guidelines.

### Statistical analyses

We ran General Linear Mixed Models (LMMs, lme4 package)^35^ to investigate temporal variation in our behavioural parameters for two reference days (D7 and D13). Both days were analyzed separately with visit rate as dependent variable and time window (corresponding with Control, Alone and Reunion), sex (Male, Female) and their interaction as explanatory variables. NestID was included as a random factor, because male and female partners of a given nest are not independent. Similar models were performed for alternation, excluding sex as an explanatory variable as this is a pair parameter.

Sex-specific and age-dependent nest return times were assessed with two separate LMMs. One LMM for the return time when the partner was caught, the other LMM for return time of the caught parent. Furthermore, we performed three LMMs to assess within-individual changes in nest visit rate and within-pair changes in their degree of alternation. First, the difference (change) in visit rate between the ‘Control’ and the ‘Alone’ period was used as a response variable. Sex, nestling age and their interaction were added to the model as explanatory variables. The length of the period when one parent visited the offspring alone (i.e. time difference between the nest return of both parents) was included as covariate, given the high variability in this ‘disturbance time’ across nests (range: 0.5–4.5 h, see further) and its potential to affect individual behavioural responses. In the second LMM, the difference in visit rate between the ‘Control’ and the ‘Reunion’ period was used as response variable. In addition to factors in the previous LMM, we added capture status (partner or individual itself was caught) to the statistical model. A third LMM was performed to test whether the difference in alternation level between the ‘Control’ and the ‘Reunion’ period depended on nestling age, sex of the caught individual and/or their interaction.

Given that often one parent was caught on D6 and the other parent on D12 (N = 15 nests), NestID was added as a random factor to all the above models to account for statistical non-independence. For the same reason, individual BirdID nested in NestID was included as random effect in the models that tested for sex-specific effects. Although not within the scope of the current manuscript, yet to statistically control for potentially influential biological factors, we always added the number of nestlings and hatch date (standardized within each year) to all the above mentioned models. The normality of residuals assumption was checked with Shapiro tests (W > 0.9) for all the above models. Nest return time and the change in visit rate from ‘Control’ to ‘Alone’ were log-transformed to meet this assumption. Two females and seven males returned the day after or abandoned the brood when they or their partner was caught. These individuals were excluded from all analyses. Means and standard errors are presented in the figures and result sections unless mentioned otherwise. Analyses were performed in R 3.2.2^36^ and all statistical output is based on full models.

## Results

### Behaviour on reference days

**Figure 1:**
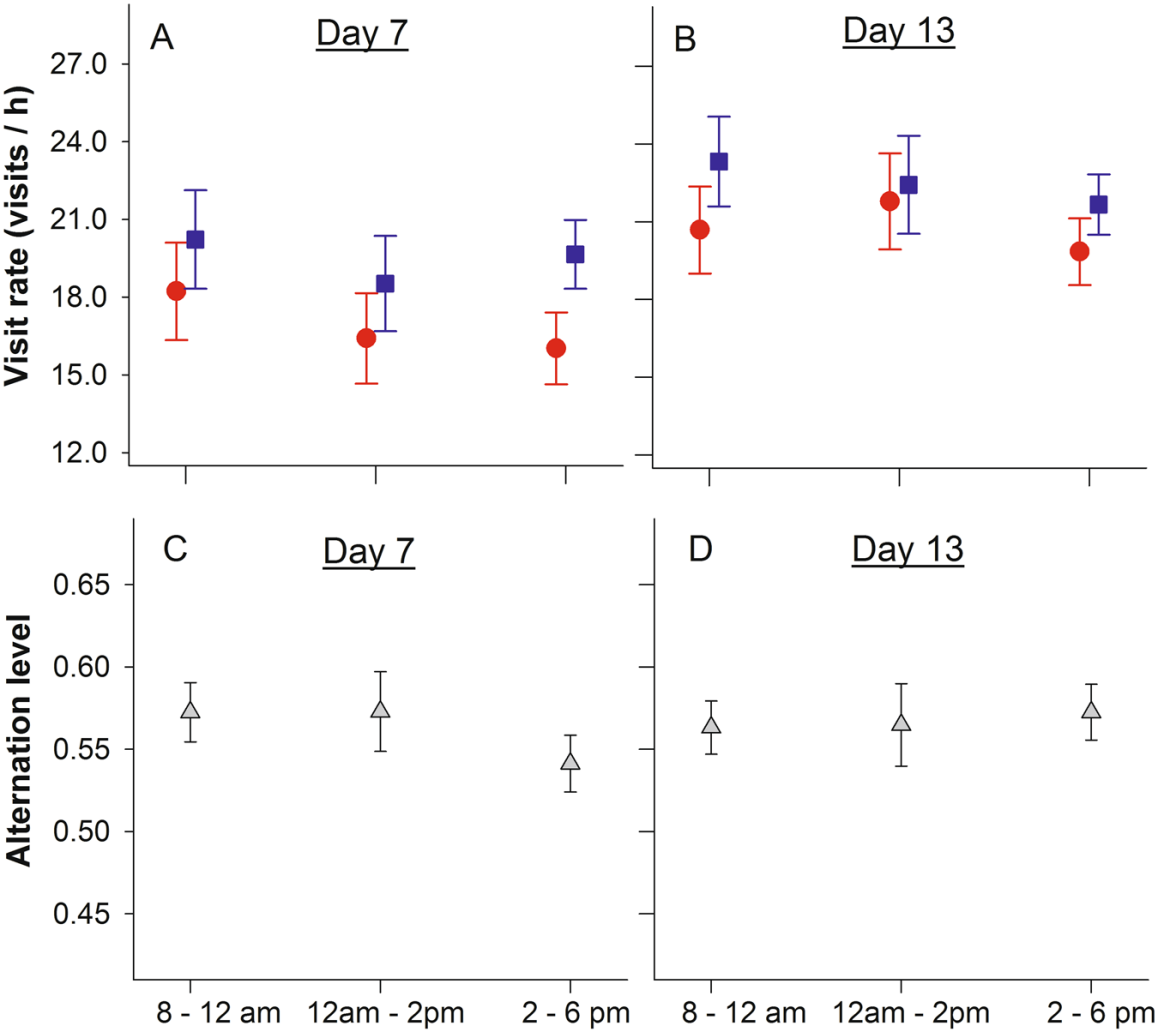
Blue tit parental feeding visits in unmanipulated conditions when nestlings are 7 and 13 days old (reference days). Nest visit rate for males (blue squares) and females (red dots) are given in panels A (day 7) and B (day 13). Panels C (day 7) and D (day 13) represent the proportion of all visits that are alternated within pairs, i.e. alternation level.

Nest visit rate did not significantly vary throughout the course of the day (day 7: F_2,100.9_ = 1.79; P = 0.17; day 13: F_2,100.9_ = 0.61; P = 0.54), a pattern that was consistent for both sexes (period*sex day 7: F_2,100.6_ = 0.41; P = 0.66; day 13: F_2,100.0_ = 0.25; P = 0.78; Fig. 1A,B). Visit rate was significantly higher in males than females when nestlings were 7 days old (F_1,103.7_ = 6.4; P = 0.012), but not when they were 13 days old (F_1,102.5_ = 1.5; P = 0.22). Visit rate increased with the number of nestlings (day 7: F_1,21.7_ = 14.1; P = 0.001; day 13: F_1,20.3_ = 7.48; P = 0.012), but did not significantly vary with hatch date on either of the two time points (day 7: F_1,24.1_ = 0.72; P = 0.40; day 13: F_1,20.3_ = 3.62; P = 0.07). The number of nestlings did not affect alternation level on day 7 (F_1,17.0_ = 0.55; P = 0.47), but had a positive effect on day 13 (F_1,17.9_ = 5.32; P = 0.033). Hatch date did not affect alternation scores on neither of the two days (day 7: F_1,16.9_ = 0.37; P = 0.55; day 13: F_1,18.2_ = 0.11; P = 0.74). The degree of within-pair coordination was consistent throughout the day (day 7: F_2,37.2_ = 1.58; P = 0.22, Fig. 1C; day 13: F_2,37.3_ = 0.08; P = 0.93, Fig. 1D).

### Nest return time

The bird that was not caught returned to the nest on average 18.9 ± 3.2 min [1.0–212.6] after its partner had been caught. This return time did not depend on nestling age (F_1,41.7_ = 0.32; P = 0.58) and was not sex-specific (F_1,47.3_ = 1.86; P = 0.18; interaction: F_1,74.8_ = 0.52; P = 0.47). Birds that were caught and returned to the nest box on the same day did so after 101.6 ± 8.6 min [32.9–264.9]. This was again independent of nestling age (F_1,49.3_ = 0.75; P = 0.39) or sex (F_1,51.5_ = 3.10; P = 0.084; interaction: F_1,61.8_ = 0.18; P = 0.68; males: 107.7 ± 10.1 min; females: 95.2 ± 14.2 min). Number of nestlings and hatch date had no effect on return times (all P > 0.10).

### Behavioural responsiveness

Both the number of nestlings and hatch date did not have a significant effect (all P > 0.10) in any of the below statistical models that explore variation in behavioural responsiveness. Neither did the duration of the disturbance, i.e. the time period when the remaining parent actually provided care alone, alter individual responses in visit rate.

**Figure 2:**
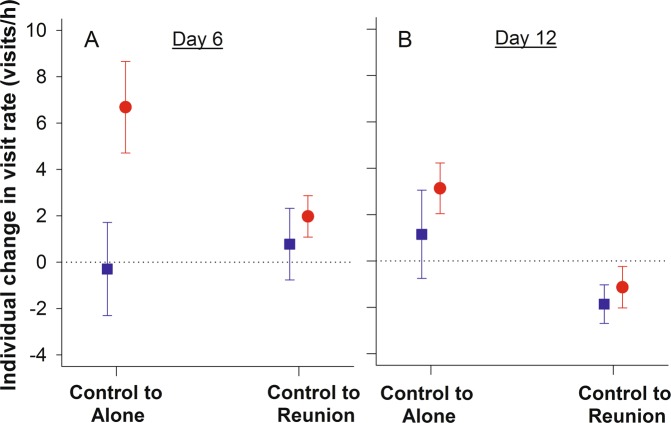
Individual change in visit rate from the ‘Control’ period relative to the period when the partner of the caught parent was ‘Alone’, and relative to the ‘Reunion’ period when the previously caught parent returned to the nest. The dotted horizontal lines indicate unaltered visit rates. The experiment was performed on day 6 (**A**) and day 12 (**B**) of the nestling period. Blue squares and red dots represent respectively males and females. Capture status (partner or individual itself) had no significant effect on the individual changes in visit rate and are therefore merged within each sex.

It took on average 1 h 42 ± 8.6 min for a caught bird to return to the nest, while its partner was left alone to feed the nestlings. During this period, we observed a clear sex-difference in responsiveness with females increasing their visit rate significantly more (from ‘Control’: 16.2 ± 0.9 visits/h to ‘Alone’: 21.0 ± 1.8 visits/hour) than males (‘Control’: 21.2 ± 1.4 visits/h; ‘Alone’: 21.6 ± 1.9 visits/hour; Fig. 2A). This sex-specific effect appeared to be slightly more pronounced with 6 days old nestlings compared to 12 days old nestlings (p = 0.056; Table 1a, Fig. 2A,B).

**Table 1:** Statistical output of three LMM’s that support Figs 2 and 3.

Effect	df	F	P
*(a) Change in visit rate from ‘Control’ to ‘Alone'*			
Nestling age	60.0	0.18	0.669
Sex	60.0	5.44	**0**.**023**
Sex × Nestling age	60.0	3.81	*0.056*
*(b) Change in visit rate from ‘Control’ to ‘Reunion'*			
Nestling age	46.7	3.96	*0.053*
Sex	35.8	0.86	0.360
Capture Status (CS)	51.9	0.74	0.395
Sex × Nestling age	45.6	0.01	0.908
Sex × CS	49.8	0.69	0.410
Nestling age × CS	60.4	0.30	0.587
Sex × Nestling age × CS	65.3	0.45	0.503
*(c) Change in alternation level from ‘Control’ to ‘Reunion'*			
Nestling age	33.0	0.02	0.890
Sex Caught	37.0	1.13	0.294
Nestling age × Sex Caught	44.8	0.00	0.999

Three covariates (number of nestlings, standardized Julian date and duration alone) did not have a significant effect (all P > 0.10) in these models (except duration alone in analysis c, see text) and are therefore not included in this table. Numerator degrees of freedom is 1 in all cases and df refers to the denominator degrees of freedom. Significant results are indicated in bold.

When the disturbed parent eventually returned, an overall increase in visit rate was found when nestlings were 6 days old (from ‘Control’: 15.2 ± 0.8 visits/h to ‘Reunion’: 17.0 ± 0.9 visits/hour; Fig. 2A), whereas an overall decrease in visit rate was observed with 12 days old nestlings (‘Control’: 20.12 ± 1.0 visits/h; ‘Reunion’: 19.1 ± 0.9 visits/hour; Fig. 2B). The parental response during this reunion period was independent of sex and capture status (Table 1b). Hence, the change in visit rate by one parent is generally matched by the change in visit rate by its partner.

**Figure 3:**
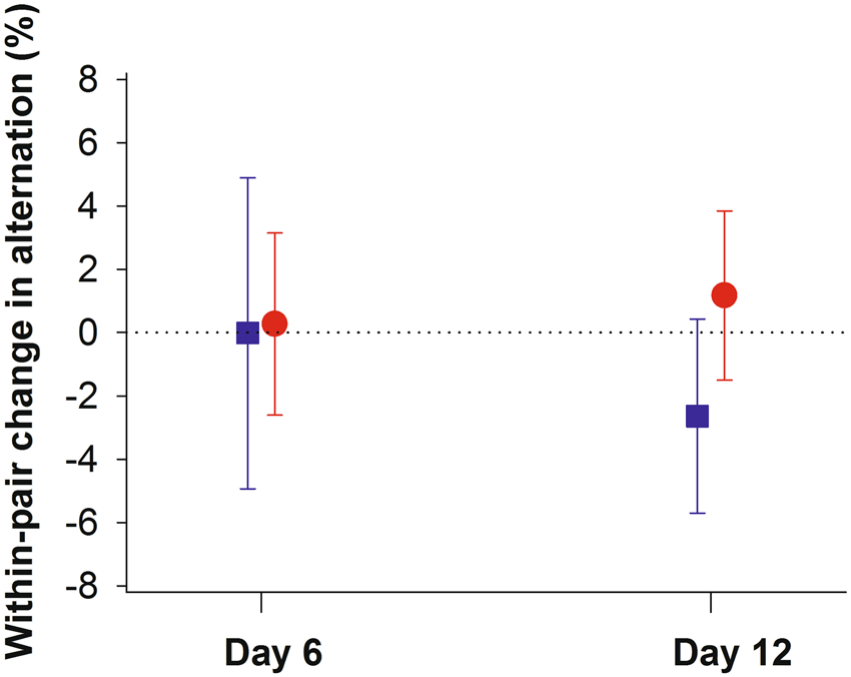
Limited within-pair changes in alternation levels from the ‘Control’ to the ‘Reunion’ period when nestlings were 6 and 12 days old. The dotted horizontal line indicates no changes in alternation in response to capturing the male (blue squares) or the female (red dots).

The change in alternation level within pairs from the ‘Control’ to the ‘Reunion’ period was very limited (‘Control’: 55.6 ± 1.4%; ‘Reunion’: 55.4 ± 1.4%; Fig. 3) and did not depend on nestling age or sex of the caught parent (Table 1c). However, a positive association was found between the change in alternation levels and the time that one parent spent caring alone (F_1,47.8_ = 4.71; P = 0.035).

## Discussion

For bi-parental species it is well known that pair members respond to changes in their partner’s parental investment^14,20^, but it was only very recently suggested that they may also co-adjust the timing and sequence of their investment bouts^15^. Parents may speed up their next nest visit when their partner has visited, but wait for the partner to feed when they themselves were last to visit, giving rise to a pattern of alternated nest visits^15,23–25,31,37^. However, experimental studies testing the drivers and the resilience of such behavioural patterns are as yet missing. By temporarily disturbing (here catching and releasing) one of both blue tit parents during two nestling stages, we studied the feeding response of each pair member after pair reunion and how that altered the level of alternation at the pair level. Intriguingly, both pair members showed a similar feeding response after reunion, despite the temporal lack of care by only one of them. Likewise, the levels of alternation resembled those observed prior to our manipulation. The implications of these findings are discussed below.

### Diurnal variation on reference days

In unmanipulated conditions male and female blue tits fed their offspring at a rather constant rate throughout the day, independent of nestling age. Such limited diurnal variation was also found for this species in a U.K. population^38^, which is in our study a prime condition that enables direct comparison between pre- and post-manipulated conditions during experimental days (see further). Yet, parental visit rates increased with nestling age, with almost 20% higher visit rates when nestlings were 13, relative to 7 days old. This is most likely driven by an increase in offspring need with age^21,31,39^. Interestingly, we found that female visit rate was significantly lower than male visit rate with seven days old nestlings, an asymmetry that disappeared when nestlings were older^23^. A likely explanation for this initial asymmetry is a parental task specialization with females allocating time to brooding, at the cost of a reduced number of feeding visits^40,41^.

Similarly, also the levels of alternated nest visits remained relatively constant between- and within both reference days. We observed that in 55% of all nest visits were alternated, which largely corresponds to observations in great tits (58%)^42^ and long-tailed tits^24^. Alternation is potentially limited by male and female task specialization and asymmetries in visit rates. Furthermore, if one parent does not show up, or its presence is not noticed, the partner is expected to delay its next visit, but not to cease feeding the offspring entirely. However, whether alternation involves such active behavioural regulation cannot be addressed with a descriptive or correlative approach. Alternation could potentially arise as an artefact from “passive turn-taking”^37^, for example when both pair members need a similar minimal amount of time to forage and deliver food to the offspring (cf. refractory period)^15^ or if male and female intervisit intervals are correlated through time^28^ (but see)^37,43^. Independent of the underlying mechanism, the fact that alternation remained fairly constant trough time indicates on average a high consistency in feeding behaviour within pairs.

### Individual response to partner absence and return

It took on average 1 h 42 min for a blue tit parent to resume provisioning behaviour after being caught. This is in accordance with a previous study in a different population of the same species (on average 1 h 54 min)^30^. The partner of the caught parent waited on average 19 minutes before resuming care for the offspring alone, which is much longer compared to an average intervisit interval of 3 minutes in undisturbed conditions. Catching itself happened quickly and handling occurred at least 15 m away from the nest, yet the partner of the caught individual was potentially also temporarily disturbed. During the following 1.5 hour alone (on average), we observed a clear sex-difference in feeding behaviour. Males were nearly irresponsive to the absence of their partner, whereas females increased their visit rate on average with 70% when nestlings were six days old and 20% when nestlings were twelve days old. Partial compensation is a common response across biparental birds following handicapping or widowing experiments^14^. Such responsiveness to the levels of partner care is thought to be one requirement for coordinated parental feeding activities, but here we found that a compensatory response was limited to females.

The observed sex-difference could be due to an asymmetry in the quality of gathered information about brood need and each other’s investment^32,44^. This because females spend a large amount of time in the nest box, cleaning and warming the poikilothermic nestlings^41,45^. Our observations are in line with the predicted hypothesis that the better informed parent (here females) should respond more strongly to changes in brood need, be less sensitive to changes in the cost of feeding and compensate more strongly for changes in partner effort^32^. In addition, given that brooding behaviour is restricted to females, they have the opportunity to shift in tasks and allocate more time into provisioning. Males however, may already work closer to their maximal provisioning rate and may therefore not have the potential to work harder. Alternatively, males are perhaps not willing to allocate more time to provisioning, e.g. due to uncertainty of paternity^46^.

When the partner eventually returned, changes in visit rate were more limited compared to the time caring alone and thus individual visit rates after the reunion were fairly similar to the period before capture. A slight (marginally significant) effect of nestling age represented an average increase of 11% in visit rate by both parents on day six and a 5% decrease on day twelve. The different sign of this response is likely explained by the difference in vulnerability between these two developmental stages. Younger nestlings may be more affected by the preceding uniparental period, causing an elevated begging response, which in turn triggers parental provisioning^11,47^. Older nestlings may have more reserves, perhaps enabling them to cope better with prolonged periods of lowered food intake, which may make it less necessary for parents to compensate. Interestingly, male and female parents responded very similar to the return of their partner on both days, and it did not matter whether an individual itself was caught or the partner. In other words, the change in visit rate from the control to the reunion period by one parent was matched by the visit rate of its partner. Matching of visit rates was also found in a previous acoustic playback experiment with great tits, in which exaggerated offspring begging signals were displayed to one target parent. The playback increased the provisioning rate of the target parent, and these elevated visit rates were subsequently matched by the unmanipulated partner^20,21^. Our results are in line herewith and further suggest that parents behave like nothing happened after the reunion. Parents matching each other was independent from what happened between capture and reunion, both in terms of duration and the sex-specific partial compensation response discussed above.

### Unaltered levels of alternation

The alternation levels following pair reunion were particularly similar to the values before the capture, a pattern consistent for both experimental days. Intriguingly, how much an individual contributed when caring alone did not affect its level of care or the degree of alternation after reunion. Neither did we observe any form of compensation by the individual that had not fed for nearly two hours, despite the long period of biased investment that likely re-enforced sexual conflict. This may suggest that the level of alternation is a unique characteristic of a pair and the easiest way for a pair to maintain their alternation level is to match each other’s visit rates. The latter is in line with previous studies showing that partners are responsive towards each other^14,20^, which is a pre-requisite for coordination. Although we cannot completely rule out that our observed matching response is driven by common environmental conditions, we carefully suggest that alternation may serve as part of a long-term ‘binding contract’ between pair members to achieve equality in investment. Such coordinated bouts of investment may hence be key for parents to ease their sexual conflict and to improve reproductive success. However, our data does not allow a distinction between the responsive, ‘active’ part of consistent alternation levels and the ‘passive’ part that merely originates as a by-product from each parent’s consistent individual feeding behaviour^28^. Thus, rather than a behavioural mechanism within pairs, alternated nest visits could merely reflect similarities in individual quality and responsiveness towards environmental conditions or to offspring demand.

### Conclusions and future directions

Both blue tit parents quickly resumed their nest visits after a nearly two-hour experimentally induced absence by one of the parents. Despite this extensive period of unequal contribution in parental care, the overall proportion of alternated nest visits did not alter within pairs. This suggests a high resilience of parental care behaviour against environmental disturbance, which may buffer against stochastic environmental effects. Intriguingly parents matched their feeding behaviour after disturbance suggesting some form of coordination, but it remains to be shown whether turn-taking acts as a driver, i.e. a behavioural mechanism for the coordination of care within pairs or whether it merely reflects analogous individual provisioning behaviour. We urge future studies on parental investment, to consider not only individual behavioural actions *per se*, but also to consider the dynamics and degree of coordination among interacting individuals. Despite the low within-pair variation, we observed substantial among-pair variation in both investment and alternation^34^, of which the causes - such as behavioural, morphological and physiological compatibility within-pairs - remain as yet unknown.

## Supplementary information

Dataset 1
